# Diffuse alveolar haemorrhage as a rare complication of antiphospholipid syndrome

**DOI:** 10.1002/rcr2.948

**Published:** 2022-04-05

**Authors:** Ishith Seth, Shyam Prakaash Bhagavata Srinivasan, Gabriella Bulloch, Dong Seok Yi, Anthony Frankel, Kelvin Hsu, Freda Passam, Roger Garsia, Tamera J. Corte

**Affiliations:** ^1^ Wagga Wagga Base Hospital Murrumbidgee Local Health District Wagga Wagga New South Wales Australia; ^2^ Bankstown Lidcombe Hospital South Western Sydney Local Health District Sydney New South Wales Australia; ^3^ South Western Sydney Clinical School University of New South Wales Sydney New South Wales Australia; ^4^ Royal Prince Alfred Hospital Sydney Local Health District Sydney New South Wales Australia; ^5^ Sydney Medical School University of Sydney Sydney New South Wales Australia

**Keywords:** antiphospholipid antibodies, antiphospholipid syndrome, APS, complication, diffuse alveolar haemorrhage, rare

## Abstract

Diffuse alveolar haemorrhage (DAH) is a rare complication of antiphospholipid syndrome. With a mortality rate of 46%, early diagnosis and management remain an ongoing challenge. Case reports are limited, and management guidelines are not yet definitive. In this case report, we present a 43‐year‐old male with DAH who required high‐dose oral steroids, intravenous methylprednisolone cyclophosphamide and rituximab over 18 months to control life‐threatening episodes of pulmonary bleeding.

## Graphical abstract

Diffuse alveolar haemorrhage (DAH) is a rare complication of antiphospholipid syndrome. With a mortality rate of 46%, early diagnosis and management remain an ongoing challenge. We report a therapeutically challenging case of treatment‐refractory DAH.

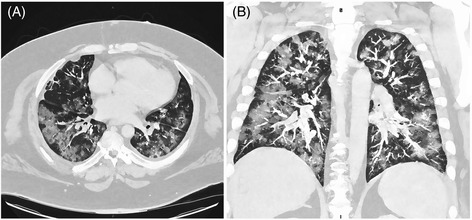

## INTRODUCTION

Diffuse alveolar haemorrhage (DAH) results from an injury or inflammation at the level of the alveolar–capillary basement membrane.^1^, ^2^ Dyspnoea, cough and haemoptysis are common presenting symptoms for DAH and bilateral ground‐glass infiltrates are common radiological findings on computed tomography (CT).^3^, ^4^, ^5^ Inflammatory markers, including C‐reactive protein (CRP) level and erythrocyte sedimentation rate (ESR), are usually elevated and alveolar fluid demonstrates neutrophilia.^6^ Systematic vasculitis is a rare, potentially fatal complication of antiphospholipid syndrome (APS) and is one of the most common causes of DAH^7^; however, many cases show capillaritis without larger vessel involvement. APS manifests with recurrent thrombotic events and 30%–40% of systemic lupus erythematosus (SLE) are positive for APS antibodies.^8^ As a result, DAH in APS is a major therapeutic challenge with patients often on lifelong anticoagulation, and sometimes requiring invasive diagnostic procedures. We report a therapeutically challenging case of treatment‐refractory DAH.

## CASE REPORT

**FIGURE 1:**
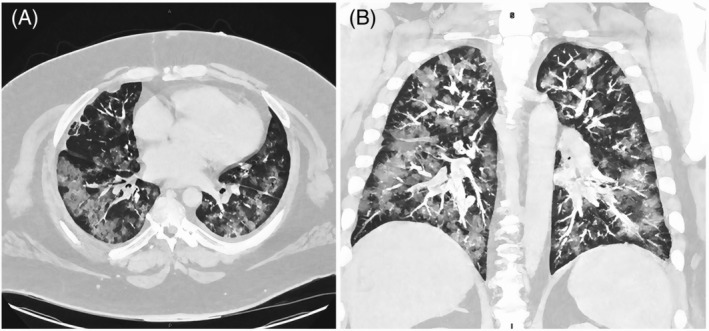
Initial computed tomography thorax illustrating diffuse ground‐glass changes with fluffy nodular appearances in February 2020. (A) Axial view. (B) Coronal view

In February 2020, a 43‐year‐old truck driver with a background of APS presented to the hospital with a 3‐day history of intermittent fevers and haemoptysis. He complained of worsening shortness of breath on exertion for 1 week. His medical history included paroxysmal atrial fibrillation, hypertension, non‐insulin‐dependent diabetes mellitus and heart failure with reduced ejection fraction. His body mass index on admission was 43.6 kg/m^2^, and he had been on long‐term warfarin for venous thromboembolic prophylaxis following a history of deep vein thrombosis (DVT) and pulmonary embolism. His blood manifested normal haemoglobin and inflammatory markers (CRP 48.7 mg/L, normal range < 3 mg/L and ESR 40 mm/h, normal range < 10 mm/h), and therapeutic levels of anticoagulation with an international normalized ratio (INR) of 2.3. He had a positive lupus anticoagulant assay, high level of anti‐cardiolipin antibodies (93 units/ml, normal range 0–13 units/ml) and abnormal beta‐2 glycoprotein‐1 antibodies (172 units/ml, normal range < 15 units/ml); however, other autoimmune and connective tissue diseases (i.e., extractable nuclear antigens, antineutrophil cytoplasmic antibodies, glomerular basement membrane immunoglobulin G, cryoglobulins, serum protein electrophoresis, double‐stranded DNA, rheumatoid factor and cyclic citrullinated peptide), as well as myositis screens (i.e., anti‐signal recognition particle and anti‐small ubiquitin‐like modifier activating enzyme heterodimer), were unremarkable. Urine analysis showed no haematuria. His initial CT images showed diffuse ground‐glass changes with fluffy nodular appearances consistent with DAH (Figure 1). Thoracic CT aortogram showed no pulmonary arterio‐venous malformations. Diagnostic bronchoscopy was unremarkable, with no endobronchial lesions or evidence of residual blood or infective causes on washings. Unfortunately, his admission to the hospital was complicated by DVT 2 days after bronchoscopy despite being on a prophylactic dose of enoxaparin before restarting warfarin. Lung biopsy was not considered safe to perform due to the risk of withholding therapeutic anticoagulation and his morbid obesity.

Despite treatment with oral prednisolone 30–50 mg daily, hydroxychloroquine 200 mg twice daily and mycophenolate mofetil (MMF) 1.5 g twice daily commencing in February 2020, the patient had three further episodes of DAH with haemoptysis, all requiring admissions to the intensive care unit over following 7 months. In September 2020, the MMF was changed to intravenous (IV) cyclophosphamide 600 mg/m^2^ monthly for 9 months and he had two 1 g induction doses of rituximab in October 2020 prompted by the recurrent episodes of DAH on MMF.

**FIGURE 2:**
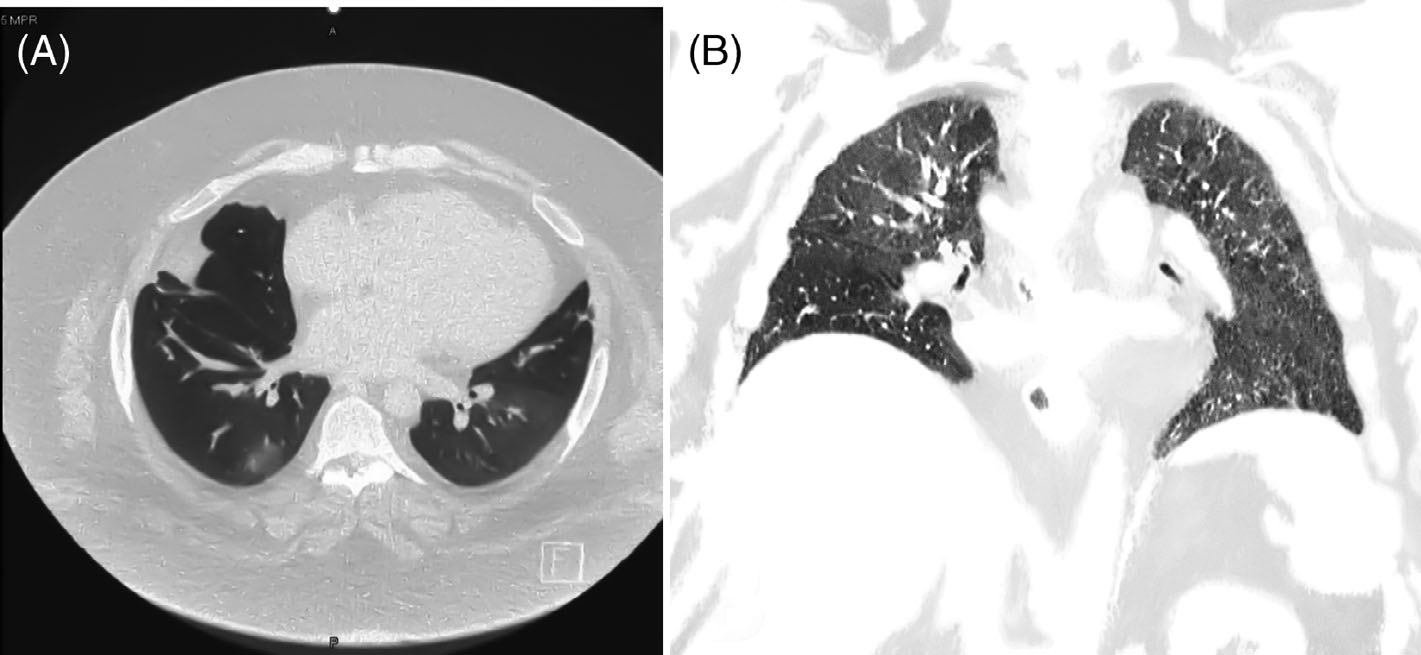
One‐year post‐initial improved computed tomography thorax, although there is still evidence of ongoing alveolar haemorrhage with bilateral ground‐glass changes. (A) Axial view. (B) Coronal view

Pulmonary infiltrates and haemoptysis were presumed to be related to pulmonary vasculitis. His breathlessness significantly improved over the next few months, was asymptomatic walking 1 km and had no further episodes of haemoptysis. His CT scan in February 2021 was improved compared to scans 12 months earlier, although there were residual bilateral ground‐glass changes (Figure 2), so the cyclophosphamide course was extended to a total of nine doses. In May 2021, he was hospitalized for biventricular failure and rapid atrial fibrillation, so his cyclophosphamide was reduced to 700 mg to mitigate cardiotoxicity risk. Following the nine doses of IV cyclophosphamide, he was restarted on MMF 1.5 g twice/daily, but 2 months later he developed worsening dyspnoea, exercise intolerance and nocturnal cough, and MMF was once again ceased as a prelude to three more scheduled monthly doses of cyclophosphamide from July 2021.

He was morbidly obese and gained 20 kg of weight during immunosuppressive treatment and developed distinct cushingoid features. His lung function in June 2021, although showing severe restriction, was unchanged since early 2020 with a forced expiratory volume in 1 s of 1.8 L (46% predicted), forced vital capacity of 2.3 L (49% predicted) and diffusing capacity of lung for carbon monoxide of 56% predicted. His cough and breathlessness were significantly better on review in August 2021. Since then, he has been on IV cyclophosphamide and prednisolone 40 mg/daily with DAH and has had a further three doses of IV methylprednisolone and 1 g rituximab. We are considering adding IV immunoglobulin (IVIG) and possibly infliximab depending on the control of disease activity.

We had not measured this patient's prothrombin time (PT) as he presented to us whilst already on lifelong warfarin which would make the interpretation of prothrombin levels problematic. However, when his prothrombin activity was investigated on admission for an episode of pulmonary haemorrhage, his coagulation profile was as follows: INR of 1.8, a PT of 25.3 s (normal range 9–13), activated partial thromboplastin time (aPTT) of 56 s (normal range 25–37), factor II level of 53% (normal range 70%–130%, consistent with warfarin effect), a dilute Russell's viper venom time ratio of 1.79, lupus aPTT ratio of 2.10 (lupus anticoagulant positive), factor VIII level of 254% (normal range 70–220) and factor XI of 123% (normal range 55–130).

## DISCUSSION

DAH is a rare and life‐threatening complication of APS.^7^ It is a small vessel vasculitis that damages the lung microvasculature by an overactive autoimmune response. Damage that ensues may involve the capillaries, arterioles and venules lining the alveoli.^9^, ^10^, ^11^ Pathological findings in DAH may include antibody‐mediated pulmonary capillaritis (neutrophilic infiltration of the alveolar wall and haemorrhage), bland pulmonary haemorrhage (alveolar spaces filled with red blood cells and the alveolar septa appears normal except for type II epithelial lining cell hyperplasia) or DAH (oedematous alveolar septa without acute inflammation).^12^, ^13^, ^14^, ^15^

In the case presented, a considerable time interval was observed between the diagnosis of APS and the first episode of DAH. Despite 6‐monthly doses of rituximab and early mycophenolate followed by IV cyclophosphamide, our patient significantly deteriorated with recurrent episodes of haemoptysis and dyspnoea. APS‐associated DAH carries a poor prognosis and only one randomized controlled trial has investigated the management of APS‐associated DAH.^16^ Current treatment goals for DAH involve: (1) supportive care including haemodynamic correction, blood transfusion and ventilatory support ranging from oxygen supplementation to high positive end‐expiratory pressure (PEEP); (2) immunosuppressive treatments to control disease activity of vasculitis; and (3) management of anticoagulation.^10^, ^17^, ^18^

The in‐hospital management of haemoptysis in concomitant APS and DAH was challenging due to bleeding risk from anticoagulation. To control bleeding, platelet counts should be maintained above 50,000/μl. Reversal or withholding of anticoagulation is often required in acute bleeding.^19^ Nebulized and systemic antifibrinolytics, such as tranexamic acid, and epsilon aminocaproic acid have been used in severe cases of DAH with varying success, but these therapies, especially fVIIa treatments, risk clotting in APS patients.^20^, ^21^, ^22^, ^23^, ^24^

High‐dose corticosteroids (500 mg to 2 g/day or 30 mg/kg/day of IV methylprednisolone for 3–5 days followed by gradual tapering over 4 weeks) are often administered to control the inflammatory activity. In refractory disease, IV cyclophosphamide can be an adjunctive immunosuppressive medication to control disease activity.^11^, ^25^ Both corticosteroids and cyclophosphamide aim to reduce acute inflammatory responses of alveolar epithelial swelling, thrombotic microangiopathy and increased production of inflammatory cells and cytokines.^10^, ^26^ Studies have validated rituximab, a B‐cell depleting anti‐CD20 monoclonal antibody, for APS‐associated DAH disease activity control.^27^, ^28^ Comparison between rituximab and cyclophosphamide therapy for DAH showed similar efficacy.^29^, ^30^ Despite following these treatment regimes in our patient, only cyclophosphamide showed clear benefit.

The most fatal complication of DAH is acute type I respiratory failure and acute respiratory distress syndrome. In this situation, high levels of inspired oxygen and PEEP are often required,^31^ where high PEEP has additional benefits in reducing active lung bleeding and preventing pneumothorax.^32^, ^33^ Immunosuppressive agents, such as MMF and hydroxychloroquine, have been shown to be effective in improving clinical outcomes in patients with APS.^34^ Fewer incidences of thrombotic complications have been recorded in patients treated with hydroxychloroquine, but it is not clear whether this is due to treatment of the SLE or the APS.^35^, ^36^

To eliminate autoreactive antibodies, plasmapheresis is recommended as adjunctive therapy.^11^ IVIG has been used as second‐line therapy in APS DAH for its immunomodulatory properties.^37^, ^38^ Infliximab, a chimeric monoclonal antibody, is another possible treatment that has been discussed in the literature; however, it lacks efficacy evidence and some literature has reported to induce DAH.^39^, ^40^ More recently, the emergence of C5a receptor inhibitor avacopan has shown promise in a randomized control trial following ANCA‐associated vasculitis patients, with a 50% reduction from baseline occurring in 86% of the avacopan/glucocorticoid group, 81% in avacopan alone group and 70% in the glucocorticoid group.^41^ Given the severity of DAH in our patient, we are considering adding IVIG and possibly infliximab or avacopan to his treatment after multidisciplinary team discussions.

Diagnosis and treatment guidance for our patient was limited to a recent review of 91 APS cases with DAH (primary or associated systemic autoimmune disease).^42^ This summarized that 11% of patients had DAH as their first presentation of APS, 55% of patients experienced recurrent disease within 35 months and mortality rate ranged between 23% and 33% due to uncontrolled DAH.^42^ Additionally, combination of cyclophosphamide with rituximab, mycophenolate or methotrexate pharmacotherapies translated to the highest remission rates (approximately 50%). IVIG and plasma exchange demonstrated similar responses (approximately 30%). These therapeutic interventions were aligned with our management, and we can attest to their relatively positive results considering the high mortality of this disease.^42^ Our case highlights the complexity of managing a patient with DAH, which required a multidisciplinary approach with no clear clinical trial based guidelines for investigation or management.

Lastly, we excluded the diagnosis of lupus anticoagulant‐hypoprothrombinaemia syndrome (LAHPS) as this patient had haemorrhage localized to the lungs with no evidence of bleeding diathesis from other systems and did not fit the coagulation profile. In particular, he never reported nose bleeds, gingival bleeding and ecchymoses, which are the most common bleeding manifestations according to Mazodier et al.'s review of 74 reported cases.^43^ Haematuria and gastrointestinal bleeding were also absent. Mazodier et al. reported median PT levels of 11% (range 1%–40%) in the 74 reported cases of LAHPS, which is inconsistent with the 53% PT observed in our patient.^43^ Therefore, this patient's clinical presentation and coagulation profile favour DAH over LAHPS, and does not support LAHPS as a diagnosis.

In conclusion, DAH should be suspected in any patient with alveolar infiltrates on chest radiograph, hypoxaemia and haemoptysis with a background history of APS. This case report presented APS‐associated DAH and symptomatic management for disease control with a clear benefit of cyclophosphamide.

## CONFLICT OF INTEREST

None declared.

## AUTHOR CONTRIBUTION

Ishith Seth, Shyam Prakaash Bhagavata Srinivasan, Gabriella Bulloch, Dong Seok Yi and Tamera J. Corte drafted the original manuscript. All authors were involved in reading, critically reviewing and approving the manuscript.

## ETHICS STATEMENT

The authors declare that appropriate written informed consent was obtained for publication of this manuscript and accompanying images.

## Data Availability

Data are available on request due to privacy/ethical restrictions.
